# CASE REPORT Papillary Fibroelastomas and the Conundrum of the Benign Intracardiac Mass

**Published:** 2012-06-15

**Authors:** Vijay A. Singh, Masood A. Shariff, Rami Michael, Basam Azab, Shani J. Fruchter, Kourosh T. Asgarian, Joseph T. McGinn

**Affiliations:** ^a^Cardiothoracic Surgery Department; ^b^General Surgery Department, Staten Island University Hospital, Staten Island, New York

## Abstract

Cardiac papillary fibroelastomas are a rare form of benign, primary cardiac tumor. They tend to develop from the valvular endocardium, with nonvalvular locations being uncommon. They are primarily found on either the mitral or aortic valve. They account for 7% of all primary cardiac tumors. Papillary fibroelastomas are usually identified through either transthoracic echocardiography or transesophageal echocardiography. The latter is more likely to provide a clearer diagnosis. Management remains controversial. The benign histology notwithstanding, the prevailing consensus is toward excision of left-sided cardiac lesions due to the risk of coronary and cerebral embolization. While the diagnosis of cardiac papillary fibroelastomas is relatively rare, the likelihood of encountering a right-sided lesion with rapid growth in a 6-month period is extraordinary. We highlight a case where an 84-year-old man with coronary artery disease was found to have a right atrial mass attached to the tricuspid valve. This mass grew by more than 1 cm in a 6-month period.

Papillary fibroelastomas (PFEs) are the second most common type of benign primary cardiac tumors.^1^^,^^2^ They are small, avascular tumors that tend to reside on the valvular endocardium.^1^^,^^3^ They are composed of a dense core of connective tissue, an intermediate layer of loose connective tissue, and a superficial layer of hyperplastic endothelial cells (Fig 1). Although these tumors are more commonly found attached to a valve on the left side of the heart, they can also be found on a right-sided valve. They are rarely noted to be attached to the atrial or ventricular wall.^4^

Most physicians elect to surgically remove left-sided tumors, due to the potential risk for embolization.^5^ Some of the direct and indirect symptoms of PFEs include syncope, angina pectoris, transient ischemic attack, stroke, myocardial infarction, pulmonary embolism, congestive heart failure, and sudden death.^1^^,^^2^^,^^6^

## CASE REPORT

This case represents an 84-year-old man with a known history of left anterior descending (LAD) coronary artery lesion with a mass noted on the tricuspid valve. He had previously undergone percutaneous coronary intervention with 2 stents placed in the LAD coronary artery, 6 years earlier. Subsequently, he was being closely followed by his cardiologist for the coronary artery lesion, when the right atrial mass was found on transthoracic echocardiography. This mass was observed for a 6-month period. A repeat echocardiogram revealed a rapid expansion of the mass. Specifically, the mass grew by 1 cm in that 6-month period. The mass was found to be fixated on the tricuspid valve. Also at this time, the patient had some complaints of dyspnea on exertion, which prompted a cardiac workup. The results of the workup eventually showed a lesion within the previously placed stent. The combination of in-stent thrombosis of the coronary stent with the rapid progression of the intracardiac mass incited the decision for surgical intervention. The patient was taken to the operating room for a single vessel coronary artery bypass grafting and excision of the intracardiac mass.

**Figure 2:**
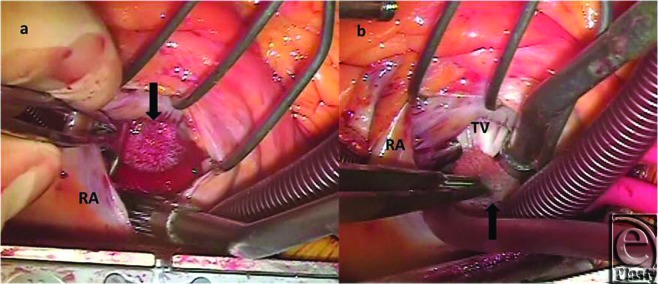
(*a*) Transaction of right atrium with a view of papillary fibroelastoma (arrow) on anterior tricuspid leaflet (*b*). RA indicates right atrium; TV, tricuspid valve; Arrow, papillary fibroelastoma.

**Figure 3:**
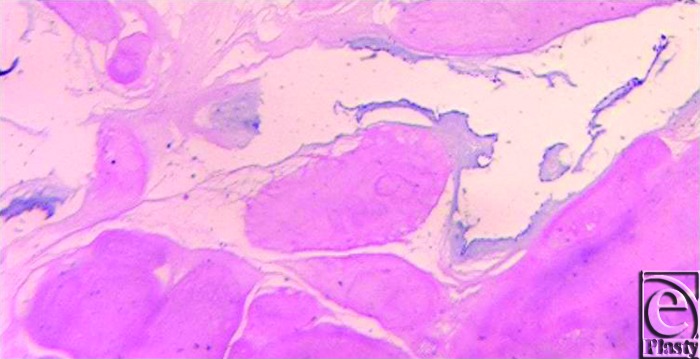
The tumor shows variably thickened avascular cores with overlying single layers of endothelial cells (hematoxylin-eosin, original magnification X2.5).

The operation was performed under normothermic cardiopulmonary bypass using ascending aortic and bicaval cannulation (Video). The right atrium was opened and a solid, well-encapsulated 1-by-2-cm mass was found to be attached to the atrial side of the tricuspid valve (Fig 2). It was subsequently shaved off and sent to the laboratory for histological evaluation (Fig 3). The right atrium was then closed, followed by the single coronary artery bypass grafting utilizing the left internal mammary artery to the LAD. After completion of the procedure, no regurgitant flow was demonstrated at the tricuspid valve. The patient was discharged 5 days after the operation, following a postoperative pneumothorax, which resolved spontaneously. The final pathology on the right atrial mass confirmed the diagnosis of PFE.[Click Here to view video]

## DISCUSSION

In general, cardiac tumors are uncommon. Cardiac PFEs account for 7% of all cardiac tumors. They are often found on the aortic or mitral valve, less commonly on the tricuspid or pulmonary valve. They are rarely found along the atrial or ventricular walls. The pathophysiology of PFEs is controversial. They have been considered to be neoplasms, hamartomas, organized thrombi, and endocardial responses to infection or hemodynamic turbulence leading to endothelial hyperplasia.^6^ Their development is also linked to iatrogenic causes, like mechanical and radiation-induced trauma.

The size of the lesion does not always correlate with the risk of developing serious morbidity. Catastrophic events can occur silently. The recorded growth of PFEs vary from 2 mm to 70 mm over a 1-year period.^2^ However, the rate of growth of these tumors have not been thoroughly analyzed in the literature. Nevertheless, the growth rate of the mass in our highlighted case far surpasses anything reported in the literature.

Papillary fibroelastomas are of clinical relevance because of their potential for embolization. If found on the left side of the heart, it can frequently lead to the patient having unclear cardiovascular and neurological embolic-like symptoms.^1^^,^^3^ Right-sided cardiac tumors remain predominantly asymptomatic until they become large enough to interfere with intracardiac blood flow, alter hemodynamic function or induce arrhythmias. This type of mass can be discovered most precisely by transesophageal echocardiography. Surgical removal of these tumors is a point of debate. The decision to operate is largely dependent on the size, location, and mobility of the mass. However, it is almost unanimously agreed upon that surgery is necessary once cardiovascular or neurological symptoms occur.^1^ Prior to surgery, the use of prophylactic anticoagulants is usually administered to decrease the risk of thrombosis formation.^1^

The unique features of our case were that this PFE occurred on the less common right side of the heart and its rate of growth was substantially faster than what is cited in the literature. As we discussed, surgical resection of any right-sided cardiac lesion is controversial. However, this case illustrates that even an initially appearing nonsurgical mass should be closely observed. If undetected, the rapid progression of the mass could have eventually caused damage to the tricuspid valve leading to hemodynamic compromise. Another reason for a close follow up relates to the sheer rate of progression. Growth of 1 cm over a 6-month period supersedes anything previously reported for other PFEs. There was fear that the mass may have a malignant component. The tissue received from the excision of the mass yielded confirmation that we were indeed dealing with a benign mass.

Benign cardiac tumors may cause significant complications regardless of its size and location. The consequences of their presence may manifest in many ways. The prudence displayed in closely observing this patient yielded an excellent outcome. This case is a good representation of the unique feature involved in the management of an intracardiac benign tumor.

Video: Excision of papillary fibroelastoma from tricuspid valve. Running time 4 mins 15 sec.

## Figures and Tables

**Figure 1:**
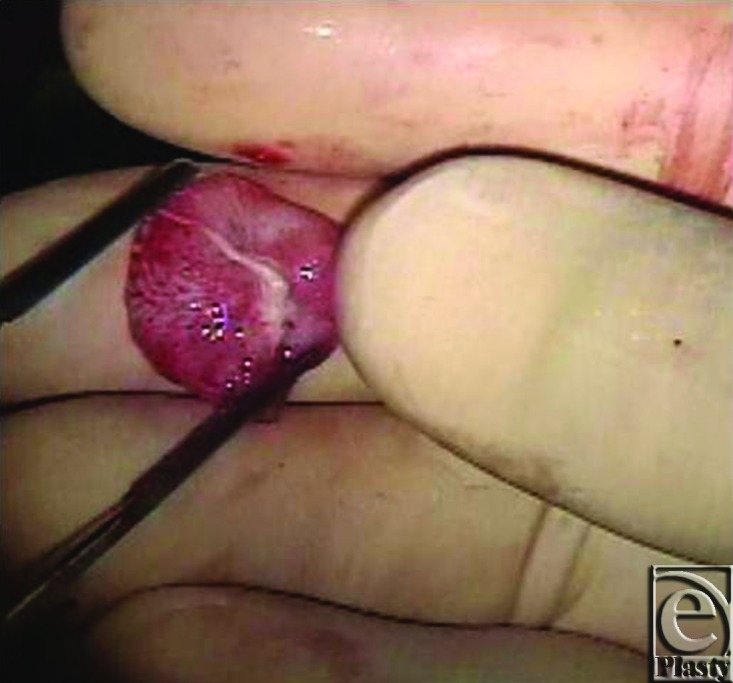
Papillary fibroelastoma.
